# Murrain of Cattle

**Published:** 1841-11

**Authors:** 


### MURRAIN OF CATTLE.

By mistake the name of Dr. John Winans was, in our last number, prefixed to an article on Murrain, written by his partner Dr. John Dawson. It arose from the name of both gentlemen being on the same sheet.

We have just received from Dr. Dawson a letter in which he makes, in reference to that disease, the following remarks:

"I have made no autopsic examinations on Bloody Murrain since I last wrote; but have made many inquiries of our farmers touching the subject. I find that the estimated *per centum* of loss by the disease, was too high, by perhaps one fourth. My views of its pathology have been confirmed. It is a general practice with those who lose cattle to take out the tallow, in doing which they expose the kidneys, which I am assured are always found "rotten." Mr. Larkins, a man whose veracity is above suspicion, says that in all the examinations he has made for 30 years, he has found the kidneys in that state, with an offensive odour." D.

